# Sucrose-induced hyperglycemia dysregulates intestinal zinc metabolism and integrity: risk factors for chronic diseases

**DOI:** 10.3389/fnut.2023.1220533

**Published:** 2023-08-11

**Authors:** Samuel Blake Mitchell, Yu-Han Hung, Trista Lee Thorn, Jiaqi Zou, Filiz Baser, Sukru Gulec, Celeste Cheung, Tolunay Beker Aydemir

**Affiliations:** ^1^Division of Nutritional Sciences, Cornell University, Ithaca, NY, United States; ^2^College of Veterinary Medicine, Cornell University, Ithaca, NY, United States; ^3^Molecular Nutrition and Human Physiology Laboratory, Department of Food Engineering, İzmir Institute of Technology, İzmir, Türkiye

**Keywords:** permeability, barrier function, zinc transporter, ZIP14, Slc39a14, glucose, enteroid, organoid

## Abstract

**Objective:**

Zinc is an essential micronutrient that is critical for many physiological processes, including glucose metabolism, regulation of inflammation, and intestinal barrier function. Further, zinc dysregulation is associated with an increased risk of chronic inflammatory diseases such as type II diabetes, obesity, and inflammatory bowel disease. However, whether altered zinc status is a symptom or cause of disease onset remains unclear. Common symptoms of these three chronic diseases include the onset of increased intestinal permeability and zinc dyshomeostasis. The specific focus of this work is to investigate how dietary sources of intestinal permeability, such as high sucrose consumption, impact transporter-mediated zinc homeostasis and subsequent zinc-dependent physiology contributing to disease development.

**Method:**

We used *in vivo* subchronic sucrose treatment, *ex vivo* intestinal organoid culture, and *in vitro* cell systems. We analyze the alterations in zinc metabolism and intestinal permeability and metabolic outcomes.

**Results:**

We found that subchronic sucrose treatment resulted in systemic changes in steady-state zinc distribution and increased ^65^Zn transport (blood-to-intestine) along with greater ZIP14 expression at the basolateral membrane of the intestine. Further, sucrose treatment enhanced cell survival of intestinal epithelial cells, activation of the EGFR-AKT-STAT3 pathway, and intestinal permeability.

**Conclusion:**

Our work suggests that subchronic high sucrose consumption alters systemic and intestinal zinc homeostasis linking diet-induced changes in zinc homeostasis to the intestinal permeability and onset of precursors for chronic disease.

**Graphical Abstract:**
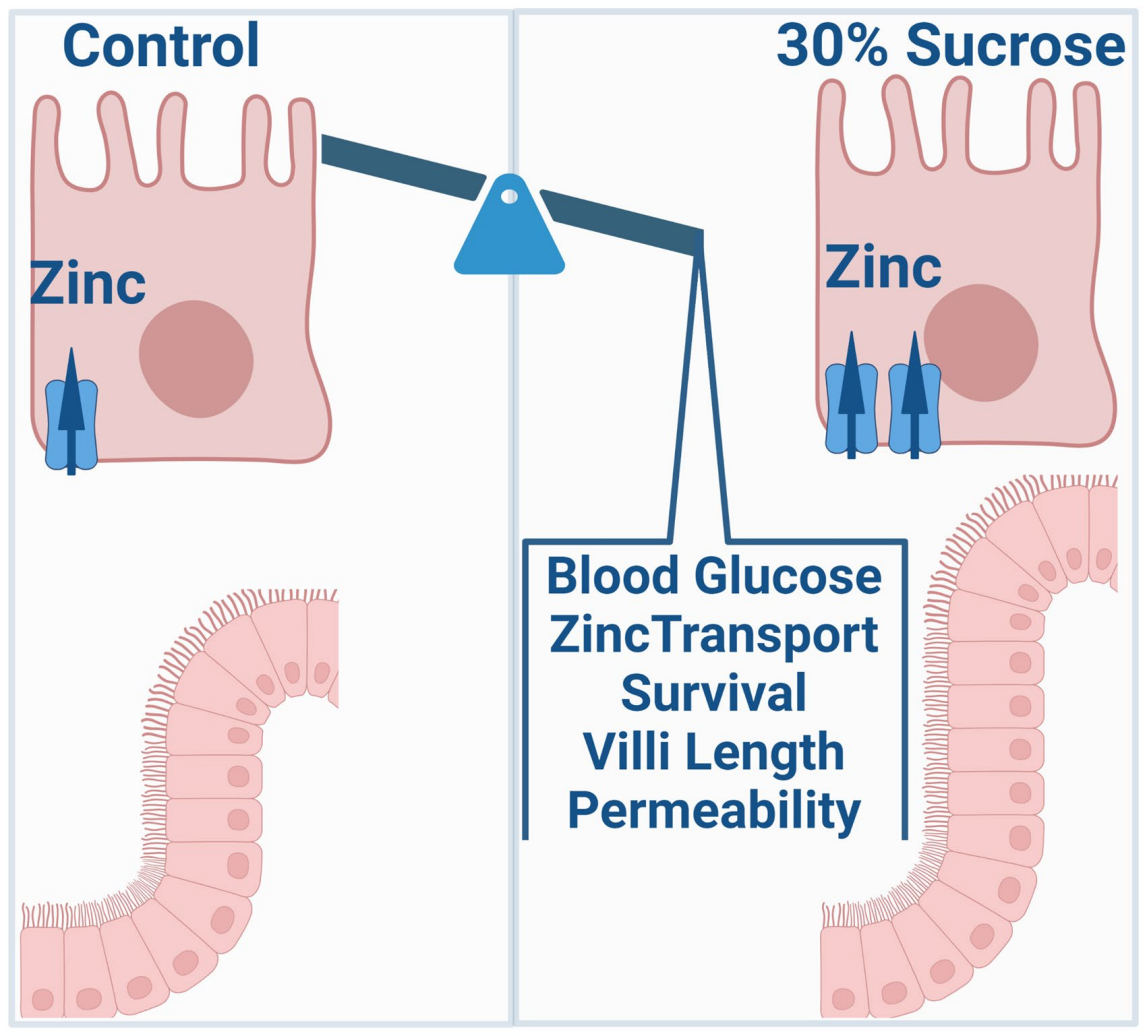


## 1. Introduction

Zinc (Zn) is an essential micronutrient with crucial catalytic, structural, and regulatory functions (1). Zn is classified as a type II nutrient since all tissues need the micronutrient Zn for cellular division and growth (2). Despite its essentiality, there is no functional reserve or storage site for Zn in mammalian systems. Mainly, the two families of zinc transporters (SLC30/ZNT and SLC39/ZIP) maintain zinc homeostasis in the body, from intestinal absorption/excretion to the distribution of Zn to the target tissues, cells, or subcellular compartments. While the zinc status regulates some zinc transporters for maintaining homeostasis, others respond to cytokines, hormones, secondary messengers, and dietary components with consequent changes in zinc distribution (3–7). Therefore, zinc metabolism is very dynamic and requires careful assessment in normal- and pathophysiology.

Zinc is essential for intestinal health. Zinc deficiency increases the risk for gastrointestinal diseases, including inflammatory bowel diseases (IBDs). IBDs, including Crohn’s disease (CD) and ulcerative colitis (UC), are chronic inflammatory disorders of the GI tract. According to a systematic analysis for the Global Burden of Disease Study, in 2017 there were 6.8 million cases of IBD globally and its prevalence is on the rise (8). The pathogenesis of IBD is not fully understood but involves a combination of genetic and environmental factors that disrupt the integrity of the intestinal epithelium (9, 10). This increases the permeability of the epithelial barrier, allowing both intestinal microbes and secreted endotoxins, such as lipopolysaccharides (LPS), to enter the circulation; a process known as metabolic endotoxemia (11, 12). Subsequent triggering of the immune response results in low-grade chronic inflammation, which can contribute to IBD development or worsen the current disease.

Furthermore, impaired intestinal barrier function and concurrent low-grade chronic inflammation are major factors contributing to obesity and diabetes, two of the most prevalent metabolic disorders whose incidence is increasing rapidly worldwide (13). Low-grade chronic inflammation activates immune reactions in metabolically important tissues such as adipose, muscle, and liver, causing insulin resistance and hyperglycemia (14, 15). A Western-type diet is characterized by a high content of refined sugar, fat, and animal proteins and has been linked to an increased incidence of chronic diseases, including type 2 diabetes, obesity, and inflammatory bowel disease (16). Clinical and experimental studies demonstrate that a high-fat diet can trigger these chronic disorders (17). Importantly, it has been shown in recent studies that dietary sugars may also contribute to preconditioning individuals for chronic disease (18, 19).

The major sugar used in processed food is sucrose, a disaccharide composed of the monosaccharides glucose and fructose. It has been shown that the consumption of sugar-sweetened beverages can alter serum levels of copper and zinc in healthy subjects (20). After consuming drinks that were sweetened by either glucose, fructose, high fructose corn syrup, or aspartate for 2 weeks, lower serum zinc levels were found in subjects who received glucose. Sucrose (30%) consumption in drinking water for 4 months lowered serum zinc concentrations in rats (21). Lumenal exposure to glucose has been known to alter intestinal permeability (22, 23), though more recent studies also have shown that hyperglycemia could be the driving factor for increased intestinal permeability (24). However, the impact of dietary sucrose on intestinal and systemic zinc homeostasis and subsequent zinc-dependent physiology remains unclear.

A western diet rich in fat and sugar is an environmental risk factor for IBD and metabolic disorders such as diabetes and obesity (17–19, 25, 26). Two common features of these chronic disorders are increased intestinal permeability and zinc dyshomeostasis. However, how high sugar consumption influences intestinal zinc metabolism and how altered zinc metabolism, in turn, affects intestinal permeability and the disease outcome has not been studied. Our results revealed that subchronic sucrose consumption altered intestinal and systemic zinc homeostasis. These alterations were concomitant with increased intestinal permeability.

## 2. Results

### 2.1. Sucrose consumption altered the metabolic phenotype in mice

**Figure 1:**
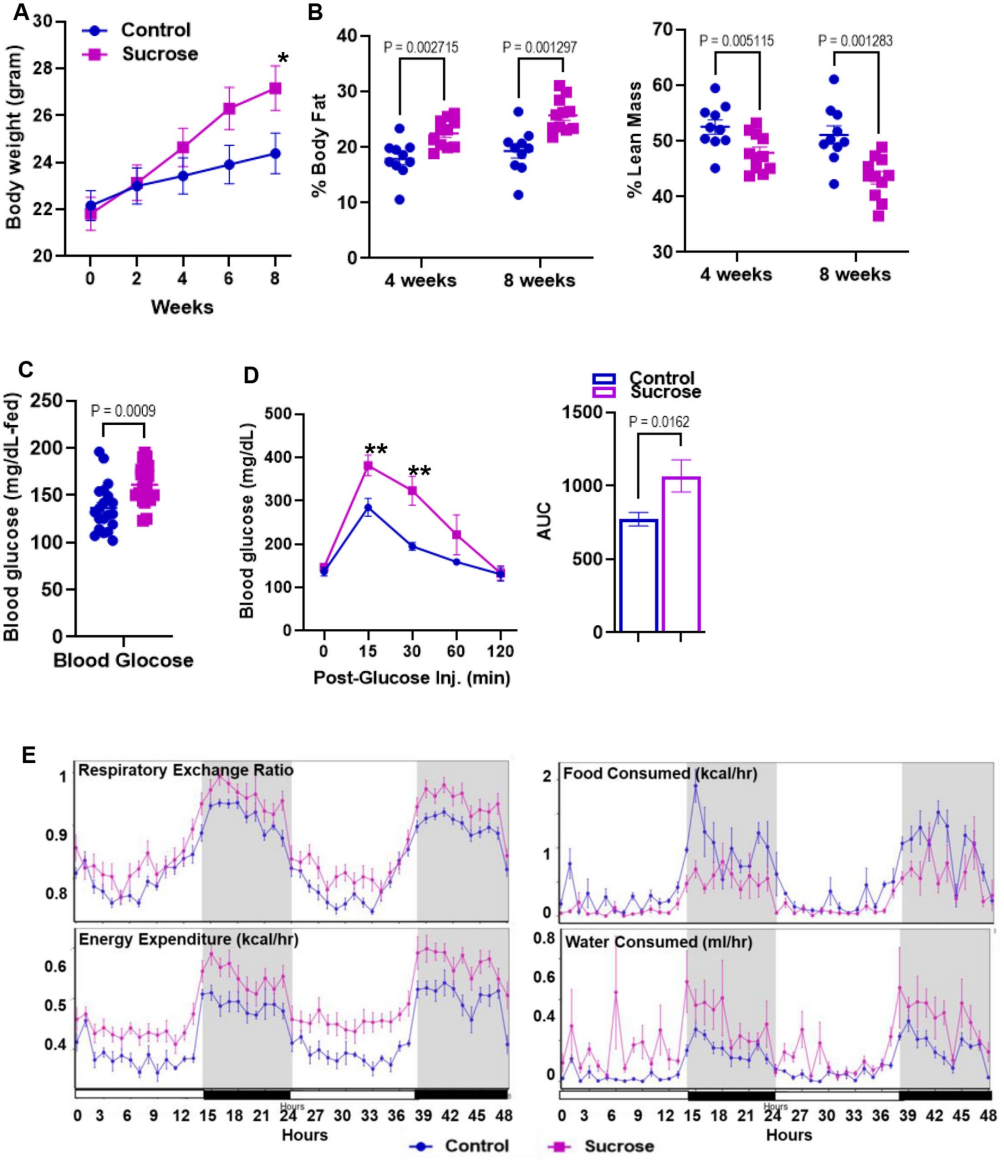
Subchronic liquid sucrose treatment induces metabolic dysregulation. Mice were given 30% sucrose in drinking water for 8 weeks. **(A)** Body weight was measured every 2 weeks up to 8 weeks. **(B)** Analysis of body composition using time-domain nuclear magnetic resonance using the Minispec LF65 Body Composition Mice Analyzer at 4 and 8 weeks of treatment. Blood glucose levels were measured by tail bleeding using the OneTouch^®^ Ultra Blood glucose monitoring system at fed-state **(C)** and at 0, 15, 30, 60, and 120 min post glucose **(D)**. **(E)** Metabolic parameters of the mice were measured by the Comprehensive Laboratory Animal Monitoring System (CLAMS) by using Promethean (Sable Systems). Following 48 h of acclimation, data were collected for 48 h. The data were analyzed using Expedata software system (v1.9.27), Macro interpreter (v2.40), and then CalR software (v.1.2). Values are means ± SEM; *n* = 4–8 per group. ^*^0.05, ^**^0.01.

High sugar treatment has been reported to cause alterations in systemic metabolism (27) and increase the risk of the development of inflammatory bowel disease (28–30). To identify the metabolic phenotype following the high sucrose treatment, we first measured body weight every 2 weeks. At the beginning of the study, the average mouse weighed 22 grams. Both groups gained weight, but after 4 weeks, we observed significantly higher body weight in the sucrose-treated group (Figure 1A). Our body composition assessments by nuclear magnetic resonance (NMR) at 4 and 8 weeks revealed greater % body fat and lower % lean mass in the sucrose-treated group (Figure 1B). The blood glucose levels were higher in the fed state of sucrose treated animals at 8 weeks (Figure 1C). We observed significantly higher blood glucose levels in the sucrose-treated group in the glucose tolerance test via intraperitoneal injection (IPGTT) (Figure 1D). We also assessed changes in energy metabolism by using Comprehensive Laboratory Animal Monitoring System (CLAMs) (Figure 1E). Sucrose-treated mice had a higher respiratory exchange ratio and energy expenditure, indicating a shift towards carbohydrates as the preferred energy source and greater overall metabolic activity. Sucrose-treated mice consumed more water and less food compared to control mice. The related bar graphs were also provided in Supplementary Figure S1.

### 2.2. Sucrose consumption alters systemic zinc homeostasis

**Figure 2:**
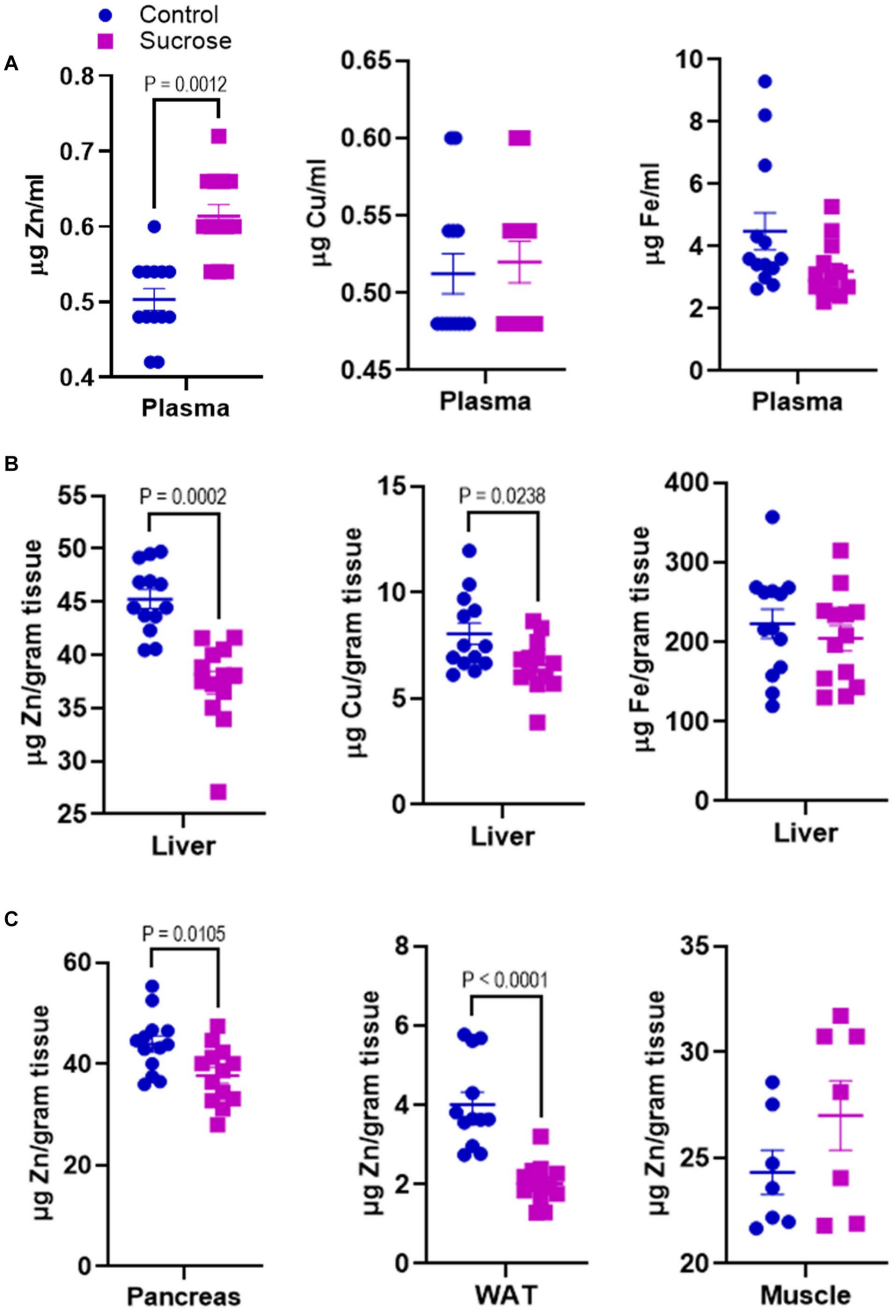
Subchronic liquid sucrose treatment alters systemic zinc homeostasis. Mice were given 30% sucrose in a drinking water for 8 weeks. **(A)** Plasma zinc, copper, and iron concentrations were measured using the microwave plasma-atomic emission spectrometer (MP-AES). Blood was collected in EDTA tubes and plasma was obtained by centrifugation. Plasma samples were diluted in deionized water (1/5) for MP-AES measurements. **(B)** Liver zinc, copper, and iron concentrations were measured using MP-AES. Liver tissues were digested in nitric acid and diluted in deionized water. Tissue weight was used as a normalizer. **(C)** Zinc concentrations in pancreas white adipose tissue (WAT) and muscle was measured using MP-AES. Tissues were digested in nitric acid and diluted in deionized water. Tissue weight was used as a normalizer. Values are means ± SEM; *n* = 8–16. Unpaired *t*-test between control and sucrose-treated groups.

Disturbances in mineral homeostasis are associated with metabolic disorders. Having seen that sucrose treatment altered metabolism in mice, we measured the concentrations of Zn, Cu, and Fe in the plasma and liver. The plasma Zn concentrations were greater in the sucrose-treated mice compared to controls, while there was no change in plasma Cu and Fe levels (Figure 2A). Liver Zn and Cu concentrations were significantly lower in the liver of sucrose-treated mice, while there was no difference in the Fe levels (Figure 2B). Since the most consistent changes were observed in Zn, we concluded that sucrose treatment had the most significant impact on Zn metabolism during 8 weeks of sucrose treatment. Then we measured Zn levels in the peripheral tissues that are closely involved with both Zn and glucose metabolism. Zn concentrations were significantly lower in the pancreas and white adipose tissue of sucrose-treated mice, and there was no difference in the skeletal muscle (Figure 2C).

### 2.3. Sucrose consumption alters intestinal zinc transport and homeostasis

**Figure 3:**
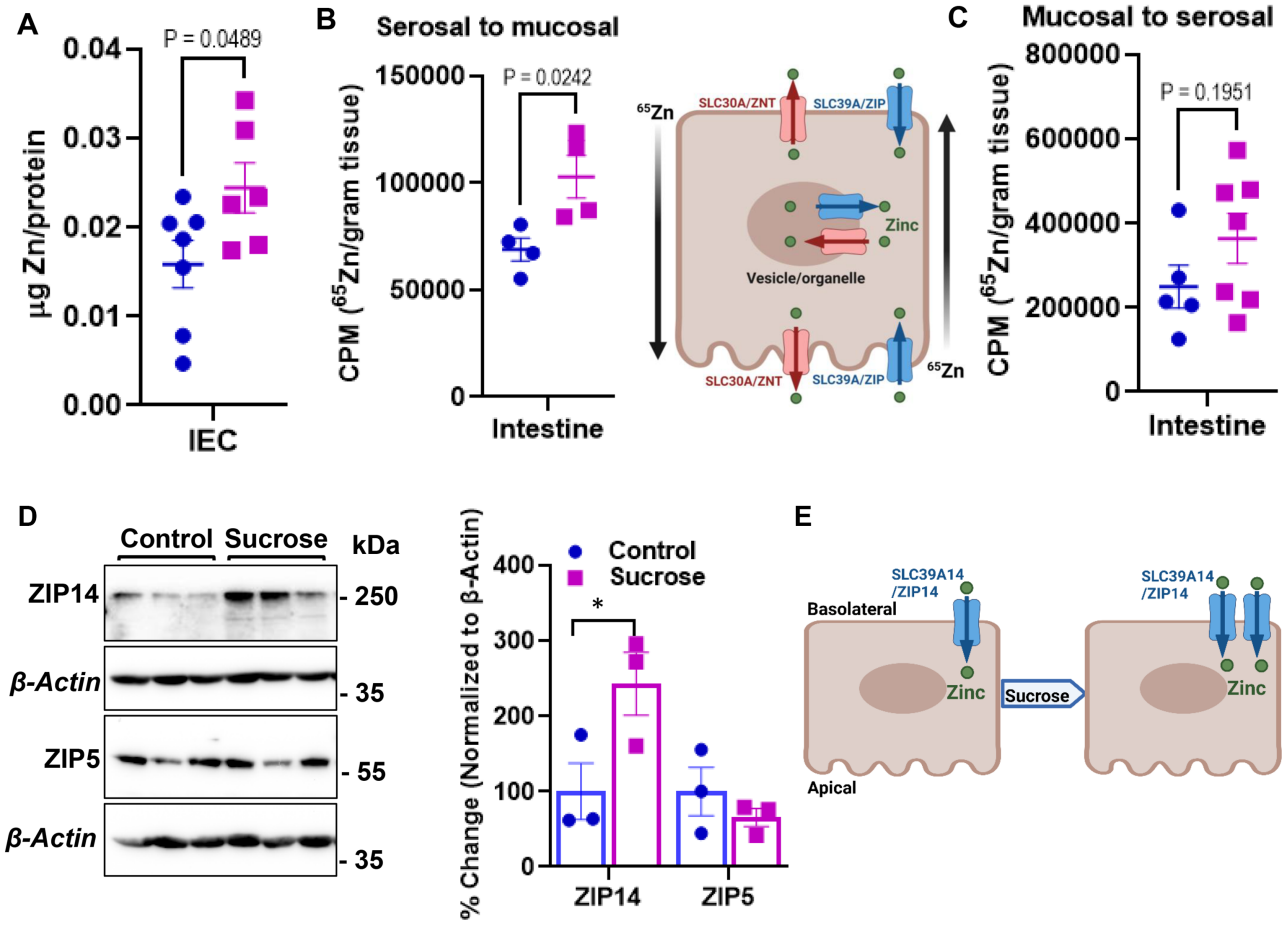
Subchronic liquid sucrose treatment alters intestinal zinc metabolism. **(A)** Zinc concentration in intestinal epithelial cells (IEC) using MP-AES. IECs were separated and digested in nitric acid. Total protein concentrations were used for normalization. **(B,C)** Following the morning fast, mice were administered ^65^Zn via either gavage **(B)** or subcutaneous injection **(C)**. Three hours later, the amount of radioactivity in intestine tissue was measured. **(D)** Representative western analyses show intestinal ZIP5 and ZIP14 protein levels from control and sucrose-fed mice (*n* = 3 mice per group). **(E)** Depiction of proposed intestinal zinc transporter and zinc regulation based on the data in **A–D**. Values are means ± SEM; *n* = 4–7. Unpaired *t*-test between control and sucrose-treated groups.

The changes in systemic Zn homeostasis led us to investigate further how subchronic sucrose consumption influences Zn metabolism in the intestine, the primary site for Zn regulation. We first measured Zn levels in isolated epithelial cells from the small intestine. We found significantly higher levels of Zn in the sucrose-treated mice (Figure 3A). To determine whether this accumulation of Zn in epithelial cells was due to increased dietary absorption or increased uptake of Zn from circulation, we administered ^65^Zn either via subcutaneous injection or gavage. There was no difference in the level of ^65^Zn in intestine tissue when administered via gavage (Figure 3C). However, ^65^Zn levels were significantly higher in the small intestines of sucrose-treated mice when administered via subcutaneous injection (Figure 3B), suggesting that the increased Zn contents in small intestine epithelial cells are at least in part due to an increase in zinc transport in the serosal-to-mucosal direction. To identify Zn transport proteins at the basolateral localization that may be responsible for the changes in Zn transport, we measured the abundance of ZIP5 and ZIP14 in the proximal small intestine, where most Zn transport occurs. ZIP14 protein expression was upregulated, and ZIP5 was unchanged (Figure 3D). The increased abundance of ZIP14, along with the ^65^Zn data, suggested that ZIP14 might facilitate the zinc transport to the intestinal epithelial cells (Figure 3E). Of note, there was no change in the Fe and Mn levels in the isolated enterocytes (Supplementary Figure S2).

### 2.4. Sucrose consumption alters intestinal homeostasis and increases intestinal permeability

**Figure 4:**
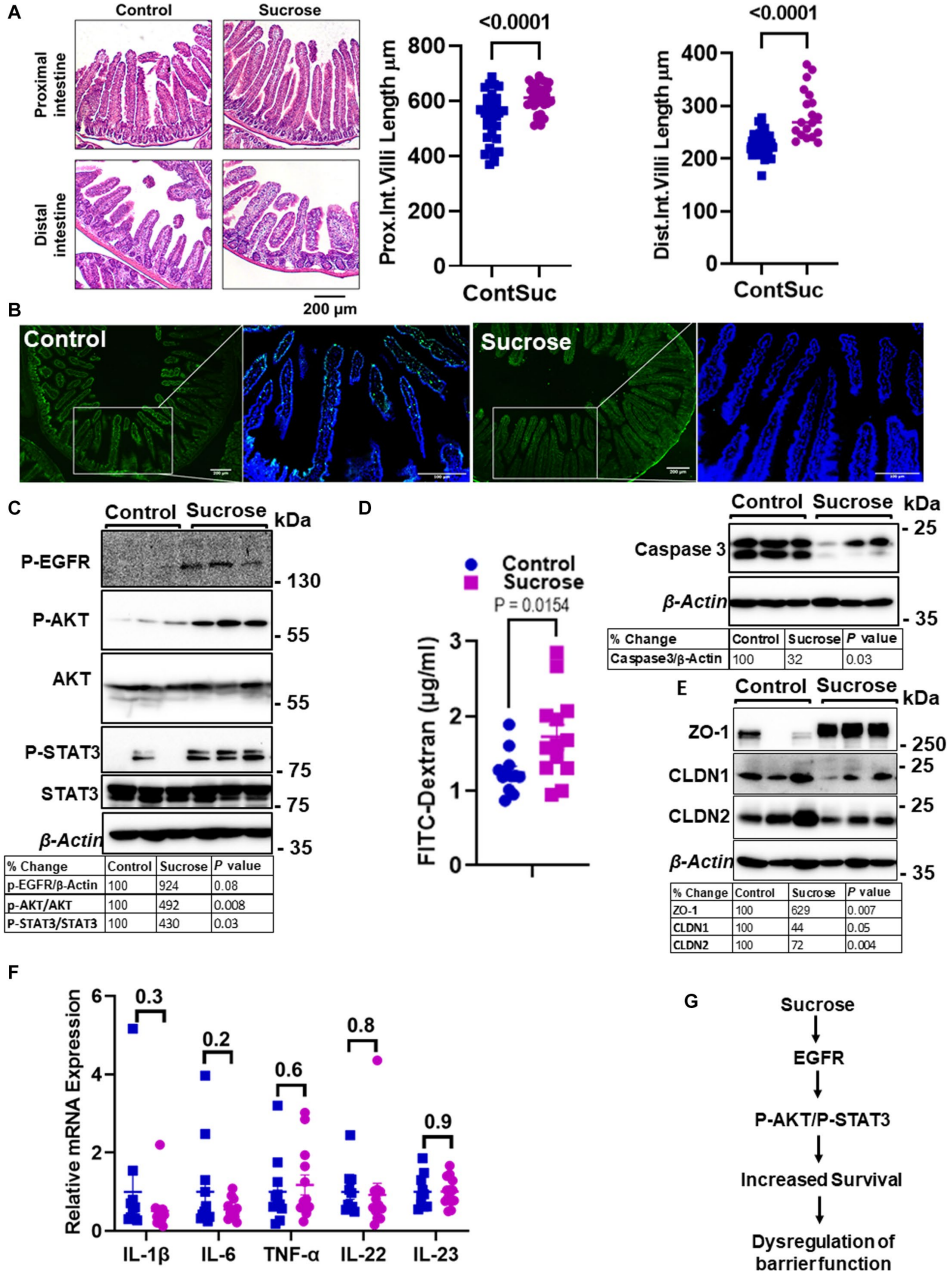
Subchronic liquid sucrose treatment dysregulates intestinal epithelial homeostasis and permeability. Mice were provided with either control or 30% sucrose-supplemented water for 8 weeks. **(A)** Representative H&E staining of the proximal and distal intestine from control and sucrose-fed mice. Villi length from both sections was quantified using ImageJ (further details are in the material methods section). **(B)** Apoptosis in the intestine tissues was assessed using the TUNEL assay (green). Representative western analyses showed intestinal caspase-3 protein levels (*n* = 3 mice per group). **(C)** P-EGFR, P-AKT/AKT, P-STAT3/STAT3, and β-actin protein levels from control and sucrose-fed mice (*n* = 3 mice per group). **(D)** Intestinal permeability was assessed by measuring plasma FITC-Dextran concentrations fluorometrically at 2 h post-gavage. **(E)** Representative western analyses showed intestinal ZO-1, CLDN1, CLDN 2, and β-Actin protein levels from control and sucrose-fed mice (*n* = 3 mice per group). **(F)** Intestine tissue cytokine expressions were measured using qPCR. **(G)** Depiction of the hypothesis. Values are means ± SEM; *n* = 3–12. The unpaired *t*-test between control and sucrose-treated groups.

Zn is essential for proper intestinal homeostasis. Therefore, having seen significant changes in intestinal Zn transport and homeostasis, we wanted to investigate how subchronic high sucrose consumption alters intestinal homeostasis further. Histological assessment revealed that sucrose treatment resulted in a significant increase in the length of the villi in both the proximal and distal small intestine (Figure 4A). The balance between the rates of proliferation and death of epithelial cells determines the intestinal villus length (31). Therefore, the increase in villi length led us to hypothesize that sucrose treatment either reduces cell death or increases cell proliferation. We found by TUNEL assay that sucrose treatment reduced apoptosis (Figure 4B). Furthermore, caspase-3 expression was lower in the intestine of sucrose-treated mice (Figure 4B). Epidermal growth factor receptor (EGFR) regulates cell proliferation and inhibition of apoptosis (32). Our results revealed an increased abundance of phosphorylated EGFR and downstream targets, AKT and STAT3 (Figure 4C), pathways associated with cell survival (33, 34).

High sugar consumption is associated with increased intestinal permeability (18, 19). Therefore, we tested whether 8 weeks of 30% sucrose treatment changes intestinal permeability. Mice were given FITC-Dextran following morning fasting, and the plasma concentration of FITC-Dextran was measured at 2 h post-FITC-Dextran gavage. We found a significantly higher amount of FITC-Dextran in the plasma of sucrose-fed mice (Figure 4D), indicating greater intestinal permeability. In the proximal intestine tissue, we found increased ZO-1 and decreased Claudin-1 (CLDN1) and Claudin-2 (CLDN2) protein expression (Figure 4E), suggesting dysregulation of tight junctions. Importantly, we did not find any significant changes in mRNA expression of *il-1β, il-6, Tnf-α, il-22, or il-23* in the intestine tissue (Figure 4F), consistent with that mice were fed a high-sugar diet (50% sucrose) (28) with no change in the cytokine expression and increased permeability. These data collectively suggested that subchronic sucrose treatment activated the EGFR-AKT-STAT3 pathway, and increased survival and permeability (Figure 4G).

### 2.5. Basolateral exposure to glucose drives dysregulation of the epithelial barrier in mouse intestine organoids

**Figure 5:**
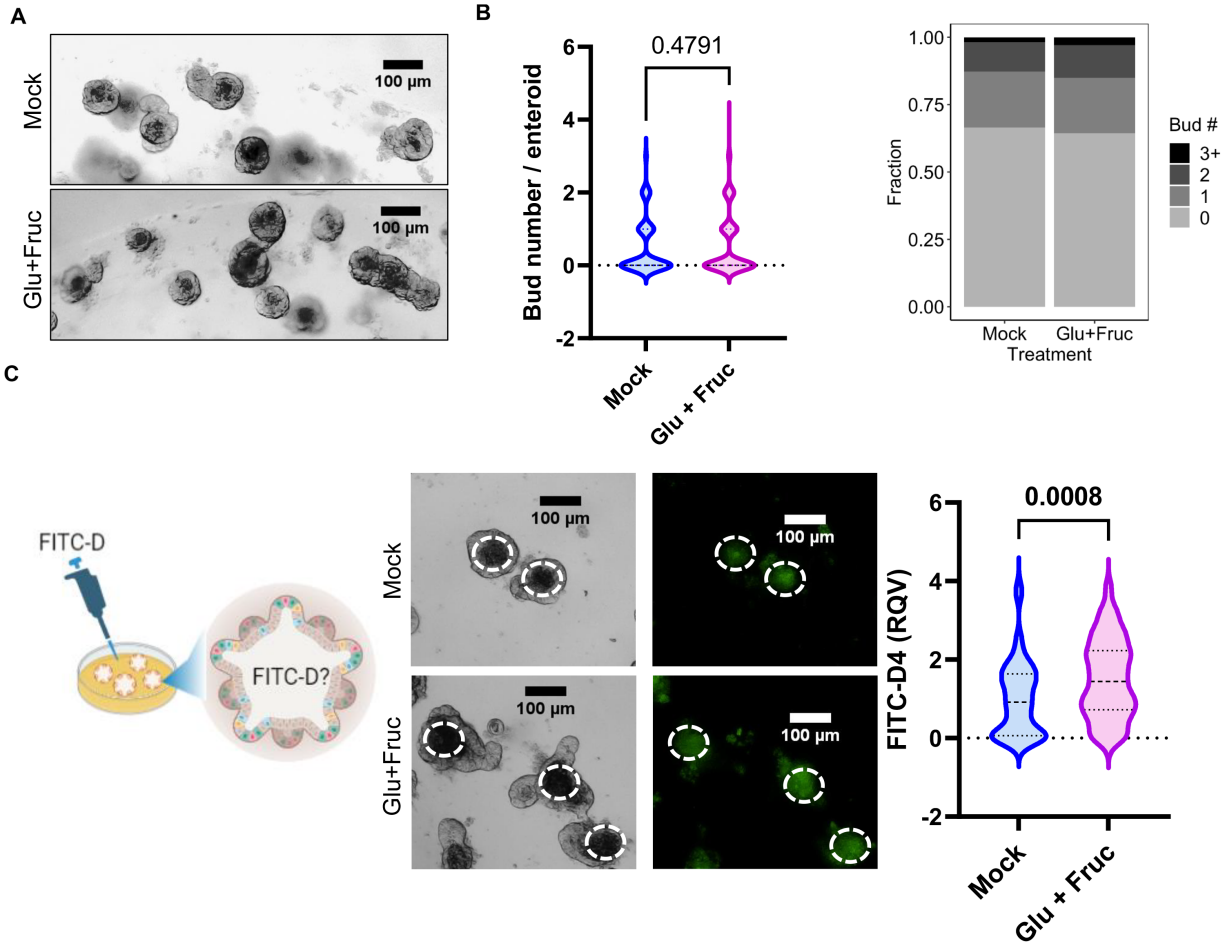
A combination of glucose and fructose treatment increases permeability in the *ex vivo* organoid system. **(A)** Representative bright field images of enteroid cultures at 24 h. post-glucose and fructose (GF) combination treatment. **(B)** Bud numbers of enteroids were quantified using the bright field images taken by OLYMPUS IX71 inverted microscope. **(C)** Representative FITC-Dextran (green fluorescence) images in enteroid cultures treated with either control or GF were acquired by OLYMPUS IX71 inverted microscope. Enteroid lumen was positioned by pairing bright field image and green fluorescence to quantify fluorescence intensity within an enteroid lumen by the software ImageJ. Values are means ± SEM; # of enteroid counted: mock, *n* = 79; GF, *n* = 111. Values are means ± SEM. The unpaired *t*-test between control and GF-treated groups.

In order to more closely study the contributions of intestine epithelial cells to the alterations in permeability and zinc homeostasis following sucrose treatment, we treated organoids derived from mouse jejunum with both glucose and fructose for 24 h. Sucrose-treated mice were hyperglycemic (Figure 1D). Furthermore, it has been shown that hyperglycemia drives intestinal barrier dysfunction (17). Therefore, we administered treatment through the media to model the basolateral exposure to glucose experienced by intestinal epithelial cells (in the organoid/enteroid system) under hyperglycemic conditions. The composition of growth media for the enteroid culture contains the epidermal growth factor (EGF), therefore mimicking *in vivo* conditions where hyperglycemia and EGFR activation coexist. To assess the proliferation of the organoids following treatment, we measured the number of buds per organoid. Combined glucose and fructose treatment did not result in altered budding of organoids (Figures 5A,B). To determine whether glucose and fructose treatment resulted in increased permeability of the organoids, we administered FITC-Dextran following treatment with glucose and fructose (Figure 5C). In agreement with our *in vivo* data, treatment with glucose and fructose resulted in increased intensity of FITC fluorescence in the lumen of organoids, indicating greater permeability.

**Figure 6:**
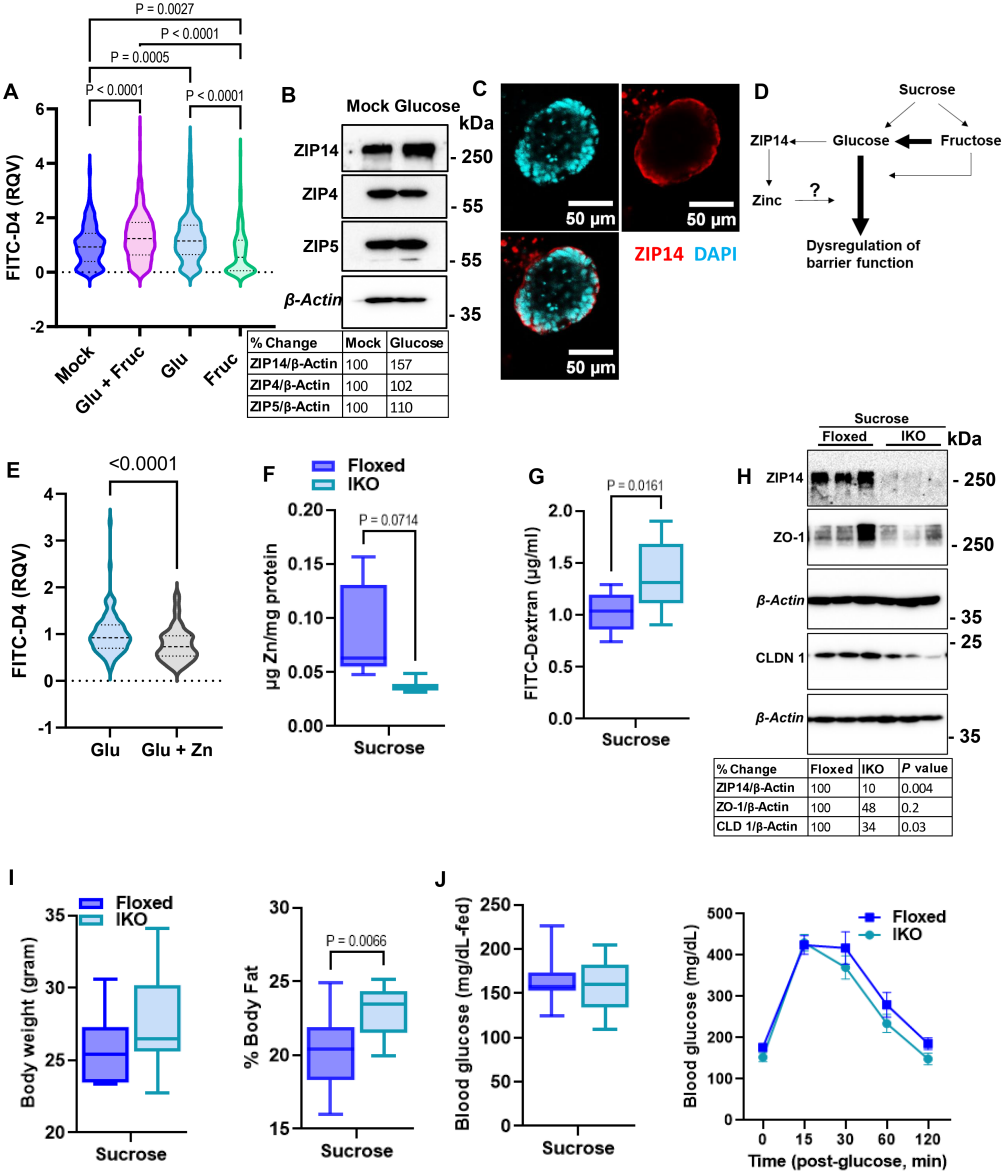
Glucose is the main driver of sucrose-induced dysregulation of intestinal epithelial barrier function. **(A)** Following 24 h of glucose (G), fructose (F), and GF treatments, FITC-Dextran (green) fluorescence intensity within the enteroid was quantified by software ImageJ. Values are means ± SEM; # of enteroid counted: mock, *n* = 322; GF, *n* = 293; G, *n* = 278; F, *n* = 253. One-way ANOVA. **(B)** Representative western analyses from two independent experiments showed ZIP14, ZIP4, and ZIP5 transporters. **(C)** Cellular location of ZIP14 at the basalateral side of the intestinal epithelium was shown in the mouse enteroid system. Images were obtained using Zeiss LSM880 confocal inverted microscope. Green: DAPI; red: ZIP14. **(D)** Depiction of the proposed mechanism of G or F-induced permeability. **(E)** Following 24 h of glucose (G) alone or combined with zinc treatments, FITC-Dextran (green) fluorescence intensity within the enteroid was quantified by software ImageJ. Values are means ± SEM; # of enteroid counted: (G), *n* = 102; G + zinc (Zn), *n* = 111. **(F)** Zinc levels in intestinal epithelial cells were measured by MP-AES. **(G,H)** Intestinal permeability was assessed by FITC-Dextran and western blot analysis for TJP. Following 8 weeks of sucrose treatment the body weight **(I)** % body fat **(J)** and blood glucose levels were measured from floxed control and intestine-specific *Zip14* KO (IKO-ZIP14 is deleted from villin-expressing cells) mice. Values are means ± SEM. *n* = 6–8 mice per group. The unpaired *t*-test between sucrose-treated floxed and IKO mice.

To determine whether glucose or fructose alone is the driver of sucrose-induced alterations in epithelial barrier function, we treated organoids with either glucose, fructose, or both for 24, and 72 h. As an indicator of organoid growth and proliferation, we quantified the number of buds per organoid and found that different types of sugar treatment did not significantly alter the bud count (Supplementary Figure S3A). However, at both 24 and 72 h, glucose treatment resulted in significantly greater permeability than fructose treatment (Figure 6A; Supplementary Figure S3B), suggesting that basolateral exposure of small intestine epithelial cells may drive the increased intestinal permeability observed in mice following sucrose treatment. Glucose treatment resulted in increased ZIP14 as in *in vivo* sucrose treatment (Figure 6B), while there was no change in the levels of ZIP4 and ZIP5. ZIP14 localization on the basolateral membrane was maintained in mouse organoids (Figure 6C). These data raised the question of whether glucose-induced increases in zinc concentrations were adaptive responses or dysregulation (Figure 6D). To test this, we treated organoids either with only glucose or glucose and supplemental zinc. Our result revealed reduced permeability with additional zinc when compared to glucose-only treated group, suggesting that the increased zinc concentration is an adaptive response (Figure 6E). In order to more closely study the impact of glucose exposure, Caco-2 cells were grown in a transwell system (35) until full confluency and were treated with high glucose (G) either basolaterally (G-BL), apically (G-Ap) or both (G-Both) for up to 48 h. Membrane resistance was measured, and changes in TEER values were plotted with time (Supplementary Figure S4A). We found the most significant reductions in TEER when high glucose was administered from the basolateral compartment. In agreement with our *in vivo* and *ex vivo* findings, *Zip14* was upregulated when glucose was administered from the basolateral side (Supplementary Figure S4B). Next, we treated Caco-2 cells with either high or low glucose media. Following treatment, high glucose-treated cells had greater total zinc content than cells grown in low glucose media (Supplementary Figure S4C). Notably, levels of Fe and Mn were unchanged (Supplementary Figure S4D).

### 2.6. Deletion of ZIP14 from intestinal epithelial cells results in greater intestinal permeability

Our *in vivo*, *ex vivo* and *in vitro* studies revealed sugar-induced upregulation of ZIP14. Furthermore, additional zinc to glucose treatment improved permeability in organoid culture. Therefore, we next tested intestinal permeability in the floxed control and intestine-specific *Zip14* KO (IKO) mice following 8 weeks of sucrose treatment. We found lower zinc concentrations in the IEC of IKO mice compared to floxed control (Figure 6F). Importantly, there was a significantly greater amount of FITC-Dextran in the plasma of sucrose-treated IKO mice (Figure 6G). We also found lower ZO-1 and Claudin1 protein abundance in the IEC from sucrose treated IKO compared to the sucrose-treated floxed mice, indicating greater permeability. We also tested metabolic parameters. There was no difference in body weight (Figure 6I) and blood glucose (in the fed state and in IPGTT) (Figure 6J), between sucrose-treated floxed and IKO mice. The only difference we observed was greater % body fat in sucrose-treated IKO mice when compared to sucrose-treated floxed mice (Figure 6I). Since there was no difference in blood glucose levels between sucrose-treated floxed and IKO mice, the result strongly suggests that the absence of ZIP14 from intestinal epithelial cells exacerbated intestinal permeability.

### 2.7. Sucrose treatment preconditions mice for intestinal inflammation, which is partially rescued by zinc supplementation

**Figure 7:**
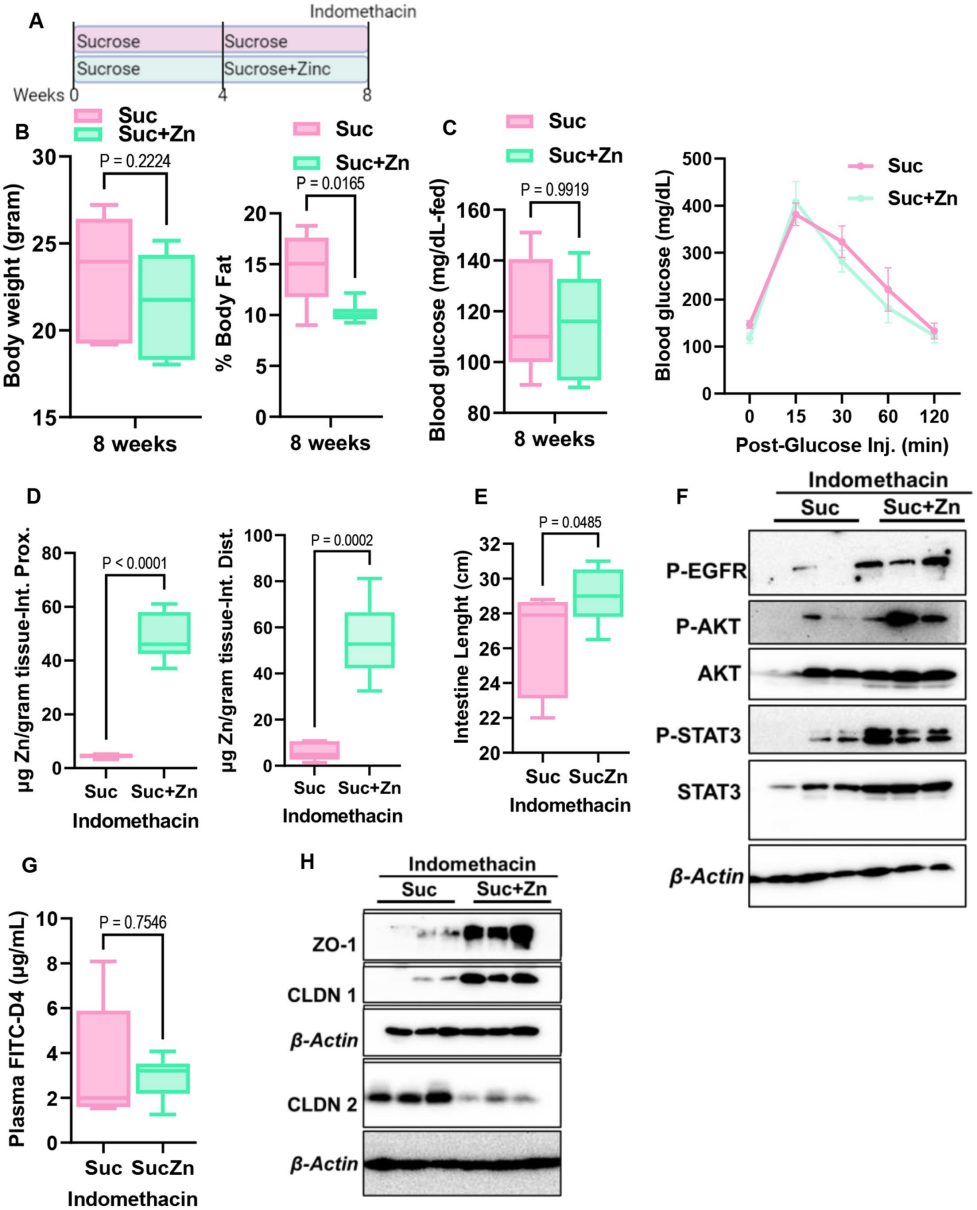
Effect of additional zinc in sucrose-fed and indomethacin-injected mice. **(A)** Mice were provided with sucrose only for 4 weeks and additional 4 weeks with both sucrose and zinc (Zn). At the end of 8 weeks, mice were injected with indomethacin for two consecutive days. **(B)** At the end of 8 weeks before indomethacin injection, body weight and percent body fat by NMR **(B)**, blood glucose levels at the fed state and following glucose injection (IPGTT) **(C)** were measured. **(D)** Following indomethacin injection, zinc concentrations in the proximal and distal intestines were measured by MP-AES. **(E)** Intestine lengths were measured at the time of tissue collection. **(F)** Western blot analysis was used to determine the protein abundance of indicated protein targets (*n* = 3 mice per group). **(G)** Intestinal permeability was measured by measuring plasma FITC-Dextran concentrations fluorometrically. **(H)** Western blot analysis was used to determine the protein abundance of indicated protein targets (*n* = 3 mice per group). Values are means ± SEM; *n* = 6–8 mice per group. The unpaired *t*-test between groups.

The development of intestinal permeability and alterations in Zn homeostasis are both closely linked to inflammatory bowel disease. Having seen that sugar treatment increases intestinal permeability and alters Zn homeostasis in mice, mouse enteroids, and Caco-2 cells, we hypothesized that sucrose treatment in mice would precondition mice for the development of intestinal inflammation. To test this, we induced small intestinal inflammation using indomethacin. Following 8 weeks of sucrose only and sucrose combined with zinc supplementation in the last 4 weeks of sucrose treatment, we administered indomethacin (5 mg/kg/day) subcutaneously on two consecutive days (Figure 7A). Before indomethacin injection, metabolic assessments showed that mice receiving Zn supplementation following sucrose treatment had a significantly lower body fat % when compared mice without Zn supplementation (Figure 7B). No changes were found when comparing body weight, fed-blood glucose levels, and blood glucose levels following glucose injection (IPGTT) (Figures 7B,C). In control experiments, indomethacin resulted in the loss of BW, shortening of the small intestine, and increased permeability (Supplementary Figures S5A–C). Using the same conditions we administered indomethacin to both sucrose alone and sucrose and Zn-supplemented groups. As expected, Zn supplementation resulted in greater Zn accumulation in the small intestine (Figure 7D). Mice that received Zn supplementation along with sucrose treatment had greater small intestine lengths (Figure 7E) along with greater amount of phosphorylated EGFR, AKT and STAT3 in the sucrose and zinc combined group when compared to only sucrose group (Figure 7F). FITC-Dextran data revealed that Zn supplementation partially rescued sucrose-induced intestinal permeability but did not reach the statistical significance (Figure 7G). Importantly when we measured tight junction protein expression in intestinal epithelial cells, we found increased abundance of ZO-1 and CLDN1 and reduced abundance of CLDN2 in mice that received sucrose along with zinc (Figure 7F).

## 3. Discussion

In this study, we provide evidence linking sugar-induced hyperglycemia to intestinal permeability and zinc dyshomeostasis, which are risk factors for inflammatory chronic diseases.

Maintaining intestinal barrier function is essential for intestinal and systemic homeostasis and overall health. The impaired barrier function has been linked to multiple gastrointestinal diseases, including inflammatory bowel disease. Similarly, zinc deficiency is reported in up to 50% of patients with inflammatory bowel diseases (26, 36, 37). However, it is unclear whether intestinal permeability or zinc deficiency are drivers or symptoms of the disease. The etiology of inflammatory bowel disease is not known, but diet is recognized as an environmental risk factor. The association between IBD development and high sugar consumption has been shown in humans (29)—furthermore, high sugar consumption preconditions mice for colitis (28, 38). Dietary sugars alter zinc homeostasis, which is essential for intestinal health. However, how high sugar consumption influences intestinal zinc metabolism and how altered zinc metabolism, in turn, affects intestinal physiology to increase the risk of developing IBD has not been studied. Our *in vivo* studies revealed that subchronic liquid sucrose consumption caused hyperglycemia, intestinal permeability, altered intestinal zinc transport, and systemic zinc homeostasis. Using *ex vivo* enteroid culture and *in vitro* Caco-2 cells, we further showed intestinal permeability and altered zinc metabolism were in part driven by basolateral glucose exposure. Mechanistically, high sugar consumption activated the EGFR-AKT-STAT3 pathway in the intestine, preventing cell death and consequent dysregulation of tight junction proteins. The sugar-induced increase in zinc transport to the intestine enhances survival.

A western diet rich in fat and sugar is an environmental risk factor for IBD (22) and metabolic disorders such as diabetes and obesity (25). It has been shown in rodent models that the mode of delivery of the sugar, rather than the amount, is the main factor in metabolic dyshomeostasis, suggesting that sugar-sweetened beverages are a potential driver of metabolic disorders. It has been shown that liquid sucrose consumption negatively affects metabolic health (7, 27). In agreement with these findings, our data revealed that consumption of liquid sucrose resulted in greater weight gain and fat/body ratio (Figures 1A,B). Furthermore, the liquid sucrose-treated group developed hyperglycemia and intestinal permeability (Figures 1C,D, 4D). Intestinal permeability shown to be driven by hyperglycemia (24), suggesting that in response to the sucrose, there were sequences of events starting with hyperglycemia leading to the intestinal permeability. Our data where enteroid cultures were treated with sugar basolaterally revealed increased permeability (Figures 5, 6), supporting that hyperglycemia, in part, could be driving impaired barrier function.

Altered systemic zinc homeostasis in response to sugar consumption was shown previously (20, 21). Here, we assessed systemic and intestinal zinc metabolism alterations related to metabolic dysregulation and intestinal permeability in response to sugar treatment. At the end of the 8 weeks of liquid sucrose treatment, we found increased ^65^Zn transport in the serosal-to-mucosal direction resulting in higher zinc levels in the intestinal epithelial cells (Figure 3). The increase in the protein abundance of basolaterally localized metal transporter ZIP14 further supported the zinc transport in serosal-to-mucosal direction. We have previously shown glucose-induced upregulation of hepatic ZIP14 (39), suggesting zinc transport in intestinal epithelial cells could be mediated by ZIP14. These *in vivo* findings were confirmed in the Caco-2 cells, where high glucose treatment increased the expression of ZIP14 and intracellular zinc concentrations (Supplementary Figure S4). These endpoint measurements raised the important question of whether alterations in intestinal zinc metabolism were a dysregulation or adaptive tissue response to high sugar consumption. Our zinc supplementation studies both *in vivo* and *ex vivo*, supported the hypothesis that zinc was needed for normal physiology.

Gut permeability has been shown to be a preclinical biomarker of Crohn’s disease onset (40). Our studies revealed that subchronic sucrose consumption caused intestinal permeability and altered intestinal zinc metabolism (Figures 3, 4). We have not found any changes in the intestinal cytokine expressions in response to sucrose consumption (Figure 4). These results are consistent with that mice were fed a high sugar diet (50% sucrose) (28) with no change in the cytokine expression and increased permeability. Similarly, mice that received 10% glucose or fructose in drinking water did not change their colon cytokine levels (38). Four months of 30% sucrose consumption in drinking water, however, increased inflammation, suggesting intestinal permeability and alterations in zinc metabolism occur before intestinal inflammation. These agree with the recent finding that intestinal permeability is associated with the later development of CD (40). Collectively, our interpretation is that high sugar consumption increases the risk of developing IBD due to increased intestinal permeability and subsequent systemic and intestinal chronic inflammation. First, high sugar consumption increases intestinal permeability due to disbalance between survival and cell death. Then, systemic and intestinal chronic inflammation is induced by the increased blood endotoxin levels resulting from intestinal leakage activating the immune system. Zinc is needed to maintain intestinal integrity and the proper function of immune cells; however, circulating zinc is depleted during inflammation. Our data with zinc supplementation suggest pretreatment may partially rescue the sugar-induced intestinal permeability. Further studies are warranted investigating the effect of zinc supplementation as a pretreatment or after disease development to test its therapeutic potential.

Chronic sucrose consumption for 16 weeks reduced serum zinc levels and increased inflammation in rats (21). We found greater serum zinc levels in the mice that received %30 sucrose for 8 weeks (Figure 2). One possible reason for the discrepancy between these studies and ours is the length of the sucrose treatment. In parallel to hyperglycemia and intestinal permeability, there could be sequential changes in zinc metabolism during subchronic sugar consumption. This possibility fits in with the fact that serum zinc levels were high along with hyperglycemia and intestinal permeability, but there was no sign of inflammation in our 8 weeks regimen. When the treatment was extended to 16 weeks, inflammation was present with lower serum concentrations (21). Similarly, low serum zinc levels and inflammation were found in IBD onset. These collectively support time-dependent alteration in zinc metabolism. This may provide important implications for the stage-dependent assessment of zinc status along with other indicators (e.g., hyperglycemia, permeability) in the development of chronic inflammatory diseases to establish better use of zinc in prevention/treatment strategies.

Zinc dyshomeostasis is closely associated with metabolic disorders such as type-2 diabetes and obesity. Hyperglycemia, inflammation, and zinc deficiency usually coexist in these chronic disorders. We previously showed inflammation-induced tissue redistribution of zinc (3, 41, 42); however, hyperglycemia-induced zinc redistribution has not been studied. The 8 weeks subchronic sucrose treatment allowed us to determine hyperglycemia-induced redistribution of zinc in the absence of inflammation. Our results revealed increased serum zinc levels and reduced liver and adipose tissue zinc concentrations. The liver and adipose tissue are metabolic organs that regulate glucose metabolism. Zinc is shown to be involved in the regulation of glucose uptake, gluconeogenesis, glycolysis, and glycogen synthesis and breakdown in the liver (7, 43). We have previously demonstrated that zinc inhibits glycogen synthesis and enhances gluconeogenesis (7). Reducing hepatic zinc might be an adaptive adjustment to limit the new glucose production and improve glycogen synthesis in subchronic high sucrose feeding without inflammation. In contrast, in high-fat diet feeding where hyperglycemia and inflammation were present hepatic zinc was increased (7). In adipose tissue, zinc is shown to be needed for the insulin response and management of the fat stores in both high-fat diet- and LPS-induced inflammation in mice (44–46). Our earlier studies found increased zinc levels in the adipose tissue in the inflammatory state. That increased zinc was needed for controlling the cytokine production in adipose tissue. Similar to the liver tissue, we found a reduction in adipose zinc levels in sucrose-treated mice without inflammation. These liver and adipose tissue findings indicated that zinc regulation differs for hyperglycemic and inflammatory states. Further studies are needed to investigate the mechanistic link between sugar consumption, zinc, and metabolic alterations in liver and adipose tissues.

Sucrose is a disaccharide composed of monosaccharides, glucose and fructose. Emerging findings by isotope tracers and metabolomics studies have shown that the small intestine metabolizes most of the dietary fructose (47, 48). Furthermore, the major portion of the tracer-labeled fructose carbons was detected as glucose in circulation, indicating that dietary fructose was converted into glucose. The spill of fructose is found in circulation when high doses overwhelm the intestinal capacity. While our *in vivo* studies used liquid sucrose treatment to model sugar-sweetened drinks, we also investigated the individual effect of glucose and fructose on permeability using the intestinal organoid system. Treatment with glucose or fructose alone induced permeability at a greater extend with the former (Figure 6; Supplementary Figure S3). Above findings on fructose metabolism may explain the difference between glucose versus fructose-induced permeability and supports the attribution that the main driver of the permeability is high glucose.

Our results revealed elongated villus length both in the proximal and distal intestine in sucrose-fed mice (Figure 4). The balance between the rates of proliferation and death of epithelial cells determines the intestinal villus length (31). Our studies revealed that sucrose treatment prevented cell death. These findings agree with the elongated villi length due to reduced cell death in the fructose-treated mice (49). Epidermal growth factor receptor regulates cell proliferation and inhibition of apoptosis (32). In the intestine, in the sucrose-fed group, we found greater activation of EGFR. Furthermore, we found higher levels of STAT3 and AKT phosphorylation in the intestine of the sucrose-treated mice. Activation of the AKT-STAT3 pathway as the downstream target of EGFR was shown (50). The intestine epithelial barrier functions as a gatekeeper at the interface between what is outside the human body and the inside. Tight junction proteins (TJPs) were dysregulated in response to sucrose treatment. Downregulation of TJPs, Claudin1, and ZO-1, was shown in multiple disease models with the signature of intestinal permeability. Consistent with these studies, we found significant downregulation of intestinal Claudin1 in response to sucrose treatment. In contrast to these studies, we found upregulation of ZO-1 despite increased permeability. To our knowledge, there is no earlier report that ZO-1 protein expression was tested in sucrose treatment. However, it was shown in Caco-2 cells that zinc treatment alone upregulated ZO-1 protein and enhanced barrier function via an AKT-dependent pathway (51). Our findings, greater zinc levels in intestinal epithelial cells, activation of intestinal AKT, and upregulation of ZO-1 from sucrose-treated mice are consistent with these earlier studies except for intestinal permeability.

The one limitation of our studies is that our data from *in vivo* studies are endpoint measurements of 8 weeks of dietary intervention. Given that 16 weeks of interventions were shown to amplify the phenotype (21), conducting 8 and 16 weeks of sugar consumption in the same study would provide a more precise comparison. Further studies are warranted to expand the time frame between sugar-induced pre-symptoms and disease onset. The second limitation of our studies is related to gut microbial changes in response to sucrose treatment. Sucrose treatments have been shown to alter the gut microbial composition and metabolome, contributing to intestinal permeability phenotype (28, 38). Our studies focused on sucrose-induced hyperglycemia altering intestinal integrity and zinc metabolism. Following *in vivo* dietary interventions, we investigated microbiome-independent glucose-regulated paths using organoid culture and *in vitro* cell lines. Further studies are needed to explore the potential contribution of the gut microbiome to hyperglycemia, intestinal permeability, and zinc dyshomeostasis in subchronic and chronic sucrose consumption.

In conclusion, our data revealed that sucrose-induced high blood glucose impairs intestinal barrier function by activating the EGFR-AKT-STAT3 pathway preventing cell death and consequent dysregulation of tight junction proteins. A sugar-induced increase in intestinal zinc functions to enhance EGFR-AKT-STAT3 signaling.

## 4. Materials and methods

### 4.1. Animals and treatments

Floxed *Zip14* (*Zip14*^*fl*/*fl*^) mice on the C57BL/6 background were generated via the targeting of introns 4 and 8 by Transposagen. The resulting *Zip14*^*fl*/*fl*^ animals were bred with B6.Cg-Tg(Vil1-cre)997Gum/J (004586, Jackson Laboratory) to create villin-expressing cell-specific KO [intestine-specific KO (IKO)] animals (47). Animal colonies were maintained using standard rodent husbandry and received a commercial, irradiated chow diet (Harlan Teklad 7912, ENVIGO, Indianapolis, IN, with 60 mg Zn/kg as ZnO) and autoclaved tap water. Age-matched mice from both sexes were used as young adults (8–16 weeks of age) for all dietary interventions. Euthanasia was through exsanguination by cardiac puncture under isoflurane anesthesia. Injections and gavage were conducted on anesthetized mice. Protocols were approved by the Cornell University Institutional Animal Care and Use Committees.

Mice were provided 30% (weight/volume) sucrose in drinking water for 8 weeks. Sucrose water was changed twice a week. In some experiments, mice received either 30% (weight/volume) sucrose alone for 8 weeks or combined with zinc treatment (48) for the last 4 weeks of the 8 weeks intervention. To induce intestinal inflammation, we injected mice with indomethacin (5 mg/kg body weight, Sigma, 17378-10G).

#### 4.1.1. Body composition

Measurements were conducted in awake animals by time-domain nuclear magnetic resonance using the Minispec LF65 Body Composition Mice Analyzer (Bruker, Germany).

#### 4.1.2. Metabolic phenotyping

Metabolic parameters of the mice were measured by the Comprehensive Laboratory Animal Monitoring System (CLAMS) by using Promethion (Sable Systems, United States). Mice were transferred to the CLAMS metabolic cages and allowed to acclimate for 2–3 days with free access to food and water. Following the acclimation, measurements of VO_2_ and VCO_2_ gas exchanges and food and water consumption were collected for 2 days.

#### 4.1.3. Fasting blood glucose

Following overnight fasting, blood glucose levels were measured by tail bleeding using a glucometer (OneTouch^®^ Ultra, LifeScan Inc.).

#### 4.1.4. Intraperitoneal glucose tolerance test (IPGTT)

Following morning fasting (4 h), blood glucose levels were measured by tail bleeding using a glucometer (OneTouch^®^ Ultra, LifeScan Inc.) immediately prior and after administration of 2 mg/kg glucose by intraperitoneal injection at 0, 15, 30, 60, and 120 min post-treatment.

### 4.2. Isolation of intestinal epithelial cells

Lumenal contents of the proximal intestine were flushed by PBS prior to everting them using bamboo sticks with pointed ends (49). Everted intestines were incubated in PBS with 1.5 mM EDTA solution for 20 min and released epithelial cells were collected by centrifugation at 4°C at 500 × g for 5 min. Following two washes, the pellets were resuspended in lysis buffer (Tris-HCl, 137 mM NaCl, 10% glycerol, 1% Triton X-100, 2 mM EDTA) with protease and phosphatase inhibitors (Thermo Scientific, 1860932 and 78428, respectively) added along with the PMSF (Sigma-Aldrich, BP-481).

### 4.3. Metal assays

Following intestinal epithelial cell isolations, cell pellets were digested at 80°C overnight in HNO_3_ to measure metal concentrations using microwave plasma-atomic emission spectrometry (MP-AES) (Agilent, Santa Clara, CA). Normalization was to total protein concentrations. Tissue metal concentrations were measured by MP-AES following overnight digestion at 80°C and appropriate dilutions for each tissue with deionized water. Normalization was to wet tissue weight. Blood was collected by cardiac puncture into EDTA tubes, and plasma was obtained by centrifugation at 3000 × g for 15 min. Following dilutions (1/5) in deionized water, plasma metal concentrations were measured by MP-AES.

### 4.4. ^65^Zn transport

Following overnight fasting, mice were administered ^65^Zn (Eckert & Ziegler, Valencia, CA) either by oral gavage (5 μCi/mouse/100 μL) or subcutaneous injection (2 μCi/mouse/100 μL). The tissue collection was at 3 h post-^65^Zn administration. Intestine tissue was perfused with a metal chelating buffer (10 mM EDTA, 10 mM HEPES, and 0.9% NaCl_2_). The amount of radioactivity in whole tissues was measured by the gamma counter and normalized by the wet tissue weight.

### 4.5. RNA isolation and qPCR

The total RNA of intestine tissues was isolated using TRI Reagent (Molecular Research Center Inc. # TR118). cDNA synthesis was performed using M-MLV reverse transcriptase (Invitrogen, #28025-013). Gene expression was measured using PowerTrack SYBR Green Master Mix (Invitrogen, #A46012). The quantitative PCR (qPCR) reactions were run in the Roche Lightcycler 480-II RT-PCR system. Primers used in this study were purchased from Integrated DNA Technologies, Coralville, IA. Primer sequences used in this study include: *il-1β* forward 5′-CAACCAACAAGTGATATTCTCCATG-3′, reverse 5′-GATCCACACTCTCCAGCTGCA-3′, *il-6* forward 5′-TAGTCCTTCCTACCCCAATTTCC-3′, reverse 5′-TTGGTCCTTAGCCACTCCTTC-3′, *Tnf-α* forward 5′-CAAAATTCGAGTGACAAGCCTG-3′, reverse 5′-GAGATCCATGCCGTTGGC-3′, *Il-22* forward 5′-ATGAGTTTTTCCCTTATGGGGAC-3′ reverse 5′-GCTGGAAGTTGGACACCTCAA-3′, *il-23* forward 5′-ATGCTGGATTGCAGAGCAGTA-3′ reverse 5′-ACGGGGCACATTATTTTTAGTCT-3′. Data were normalized with Rplp0 measurements for relative quantitation. Rplp0 forward 5′-AGATTCGGGATATGCTGTTGG-3′ reverse 5′-TCGGGTCCTAGACCACTGTTC-3′.

### 4.6. Histology

Animals were transcardially perfused with ice-cold 1x PBS. The intestines and colons were removed and fixed in formalin overnight at 4°C. Intestines were cut in half, proximal and distal. Both intestines and colon were cut into 1 cm pieces and collected into gut bundles (50). They were then placed in sucrose gradients (10, 20, 30%) until tissues sunk. Gut bundles were frozen in OCT (4583, Sakura) and cryosectioned at 10 μm thickness. The sections were stained with H&E (Abcam). All sections were longitudinal.

### 4.7. Histological analysis

Images of tissue sections were taken using the OLYMPUS IX71 microscope. Approximately 10 villi and 10 crypts from each animal were measured using ImageJ to determine villi length and crypt depth (44). Villi length was measured from base of villi to villi tip. Crypt depth was measured from base of crypt to mouth of crypt. Complete villi and crypts were chosen for this purpose. The measurements were pooled for each group (*n* = 3) and t-tests were performed (51). Crypt density was determined by calculating the number of crypts per mm-1 along submucosal circumference (51).

To conduct TUNEL staining, sections were stained with TACS 2 TdT-Flour *In Situ* Apoptosis Detection Kit (R&D Systems, 4812-30-K) according to manufacture’s protocol. To visualize TUNEL-positive cells, 4× and 10× images of tissue sections were taken using the OLYMPUS IX71 microscope (Melvile, NY).

### 4.8. Western blotting

All the buffers were supplemented with protease inhibitors (AGScientific) during enterocyte separation. Solubilized proteins were separated by 10% SDS-PAGE. Visualization was by chemiluminescence (SuperSignal, Thermo Fisher, 34580) and digital imaging (Protein Simple, San Jose,). Rabbit anti-mouse ZIP14 antibody was custom-made by Genscript (Piscataway, NJ) (47). Β-Actin (Cell Signaling, 8457), Claudin 1 (ThermoFisher, 71-7800), Claudin 2 (Cell Signaling, 48120), ZO-1 (ThermoFisher, 61-7300), P-EGFR (Cell Signalling, 3777), P-AKT (Cell Signalling, 4060), AKT (Cell Signalling, 4691), P-STAT3 (Cell Signalling, 9145), STAT3 (Cell Signalling, 30835), Cleaved Caspase 3 (Cell Signalling, 9661), SLC39A4/ZIP4 (Proteintech, 20625-1), SLC39A5/ZIP5 (Novusbio, NBP3-04949). Band intensities were measured by the software ImageJ. Values for protein of interests were normalized to their control (pan-proteins or β-actin). The percentage of changes over control groups was presented.

### 4.9. Isolation of mouse jejunal crypts

Jejunal crypts were isolated from female floxed mice (8–12 weeks old) as previously described (51). Small intestine was excised from mice and divided into three equal segments. The jejunal segment (the middle segment) was processed for crypt isolation. Subsequent to luminal flushing with ice-cold PBS, the tissue was longitudinally cut and subjected to incubation in 3 mM EDTA in ice-cold PBS with 1% (v/v) Primocin^™^ (InvivoGen, ant-pm-1) for 15 min at 4°C. The mucosa of the intestinal pieces was gently scrapped of mucus, shaken in ice-cold PBS with 1% (v/v) primocin for 1 min, and incubated in fresh 3 mM EDTA in ice-cold PBS with 1% (v/v) primocin for 35 min at 4°C. The intestinal pieces were then shaken in ice-cold PBS with 1% (v/v) primocin for 2 min and filtered with a 70 μm cell strainer. The resulting purified crypts were pelleted with centrifugation at 110 × g for 10 min at 4°C. The isolated crypts were used for enteroid culture.

### 4.10. Mouse enteroid culture

Mouse enteroid culture was performed as previously described (50). The isolated jejunal crypts (day 0) were placed in Matrigel (density 500–700 crypts per 5 μL Matrigel dome) and grown in reduced growth factor Matrigel (Corning, 356231) and advanced DMEM/F12 (Gibco, 12634-028) containing GlutaMAX (Gibco, 35050-061), Penicillin-Streptomycin (15140122, Gibco), HEPES (Gibco, 15630-080), N2 supplement (Gibco, 17502-048), 50 ng/μL EGF (R&D Systems, 2028-EG), 100 ng/μL Noggin (PeproTech, 250-38), 250 ng/μL murine R-spondin (R&D Systems, 3474-RS-050), and 10 μM Y27632 (Enzo Life Sciences, ALX270-333). Cell culture media was changed every 2 days. To assess the impact of dietary sugar on enteroid culture, dietary sugar supplementation was treated daily (since day 1) for the following experimental groups: (1) glucose (G-10 mM), (2) fructose (F-10 mM), and (3) glucose and fructose (GF-5 mM each). To examine the effects of zinc supplementation on enteroid culture, enteroids were treated daily (since day 1) with ZnCl_2_ at a final concentration of 10 μM. The enteroids from 24 h, 48 h or 72 h post-treatment were fixed in 2% paraformaldehyde for imaging analyses or collected for RNA/protein analyses. To assess enteroid growth, bud numbers of enteroids were quantified using the bright field images taken by OLYMPUS IX71 inverted microscope (Melvile, NY).

### 4.11. Enteroid permeability assay

The permeability of enteroids was examined using FITC-Dextran (4 kDa). Briefly, enteroids were washed with PBS and treated with FITC-4 kDa (Sigma, FDH1G) at a final concentration of 1 mg/mL for 30 min at 37°C. The enteroids were then washed by PBS and fixed in 2% PFA. The fixed FITC-4 kDa treated enteroids were processed through immunostaining procedures (described in the below “whole-mount enteroids immunostaining and imaging”) to remove extra FITC-4 kDa that is not present in the enteroid lumen. Paired bright field (for positioning enteroid lumen) and green fluorescence images (for visualizing FITC-4 kDa signal) were acquired by OLYMPUS IX71 inverted microscope (Melvile, NY). Fluorescence intensity within an enteroid lumen was measured by the software ImageJ. Only enteroids of which the luminal areas were well-focused in an image were used to measure FITC-4 kDa intensity.

### 4.12. Whole-mount enteroids immunostaining and imaging

The immunostaining of enteroids was performed as previously described (50). Briefly, the fixed enteroids were permeabilized with 0.5% (v/v) Triton X-100/PBS, washed by PBS containing 0.1% (w/v) BSA/0.02% (v/v) Triton-X/0.05% (v/v) Tween 20, and blocked with 10% (v/v) normal goat serum. Primary antibodies were used to stain ZIP14 (at a final concentration 8 μg/mL; GenScript; Piscataway, NJ). The staining was visualized by fluorescent-conjugated secondary antibody (Alexa Fluor 594, A11012; 1:1000; Thermo Fisher Scientific). Nuclei were stained with DAPI (1:10000 dilution from 5 mg/mL stock). The immunofluorescent images were acquired by OLYMPUS IX71 (Melvile, NY) inverted microscope or Zeiss LSM880 confocal inverted microscope (White Plains, NY). Images were analyzed by the software ImageJ.

### 4.13. Statistical analyses

Data are presented as means ± SEM. Significance was assessed by student’s *t*-test and ANOVA for single and multiple comparisons, respectively. Statistical significance was set at *p* < 0.05. Analyses were performed using GraphPad Prism (version 9.4.1).

## Data availability statement

The raw data supporting the conclusions of this article will be made available by the authors, without undue reservation.

## Ethics statement

The animal study was approved by Cornell University Institutional Animal Care and Use Committee. The study was conducted in accordance with the local legislation and institutional requirements.

## Author contributions

TA conceived and designed research, drafted the manuscript, and edited and revised manuscript. TA, SM, Y-HH, TT, JZ, and FB performed experiments. TA, Y-HH, SM, TT, and SG analyzed data. TA, Y-HH, SM, and SG interpreted results of experiments. TA, SM, Y-HH, TT, JZ, and SG prepared figures. TA, Y-HH, SM, TT, JZ, FB, and SG approved the final version of the manuscript. All authors contributed to the article and approved the submitted version.

## Funding

This project was supported by Cornell University Division of Nutritional Sciences funds to TA and; the National Institutes of Health under award T32-DK007158 to SM. The content is solely the responsibility of the authors and does not necessarily represent the official views of the National Institute of Diabetes and Digestive and Kidney Diseases (NIDDK) or the National Institutes of Health. NYSTEM C029155 and NIH S10OD018516 for the Zeiss LSM880 microscopes (i880 and u880).

## Acknowledgments

The authors acknowledge the staff members from Cornell Animal Facility and the Center for Animal Resources and Education (CARE) for technical support of mouse colony maintenance. The authors thank Meghan Mary Trumbull-Kennedy for technical assistance with intestinal epithelia isolation. Imaging data were acquired through the Cornell Institute of Biotechnology’s BRC Imaging Facility (RRID:SCR_021741), with NYSTEM (C029155) and NIH (S10OD018516) funding for the shared Zeiss LSM880 confocal/multiphoton microscope. Graphical abstract was generated by using BioRender.

## Conflict of interest

The authors declare that the research was conducted in the absence of any commercial or financial relationships that could be construed as a potential conflict of interest.

## Publisher’s note

All claims expressed in this article are solely those of the authors and do not necessarily represent those of their affiliated organizations, or those of the publisher, the editors and the reviewers. Any product that may be evaluated in this article, or claim that may be made by its manufacturer, is not guaranteed or endorsed by the publisher.
