# Supplementary material for: Sucrose-induced hyperglycemia dysregulates intestinal zinc metabolism and integrity: risk factors for chronic diseases
Source: Front Nutr. 2023 Aug 11;10:1220533. doi: 10.3389/fnut.2023.1220533 (PMC10450956; doi:10.3389/fnut.2023.1220533)
Supplement: Supplementary file 1 [file Data_Sheet_1.docx]

**Sucrose-induced hyperglycemia dysregulates intestinal zinc metabolism and integrity: risk factors for Inflammatory chronic diseases**

Samuel Blake Mitchell^1#^, Yu-Han Hung^1#^, Trista Lee Thorn^1#^, Jiaqi Zou^1^, Filiz Baser^2^, Sukru Gulec^2^, Celeste Cheung^1^, Tolunay Beker Aydemir ^1*^

^1^Division of Nutritional Sciences, Cornell University, Ithaca, NY, USA.

^2^ Molecular Nutrition and Human Physiology Laboratory, Department of Food Engineering, İzmir Institute of Technology, Urla, İzmir, Turkey

^#^Equally contributed

^*^Corresponding author

**Correspondence:** Tolunay B. Aydemir, Address: 225 Savage Hall, Ithaca, NY, USA.

E-mail: [tb536@cornell.edu](mailto:tb536@cornell.edu), Phone: +404 644 6919

1.
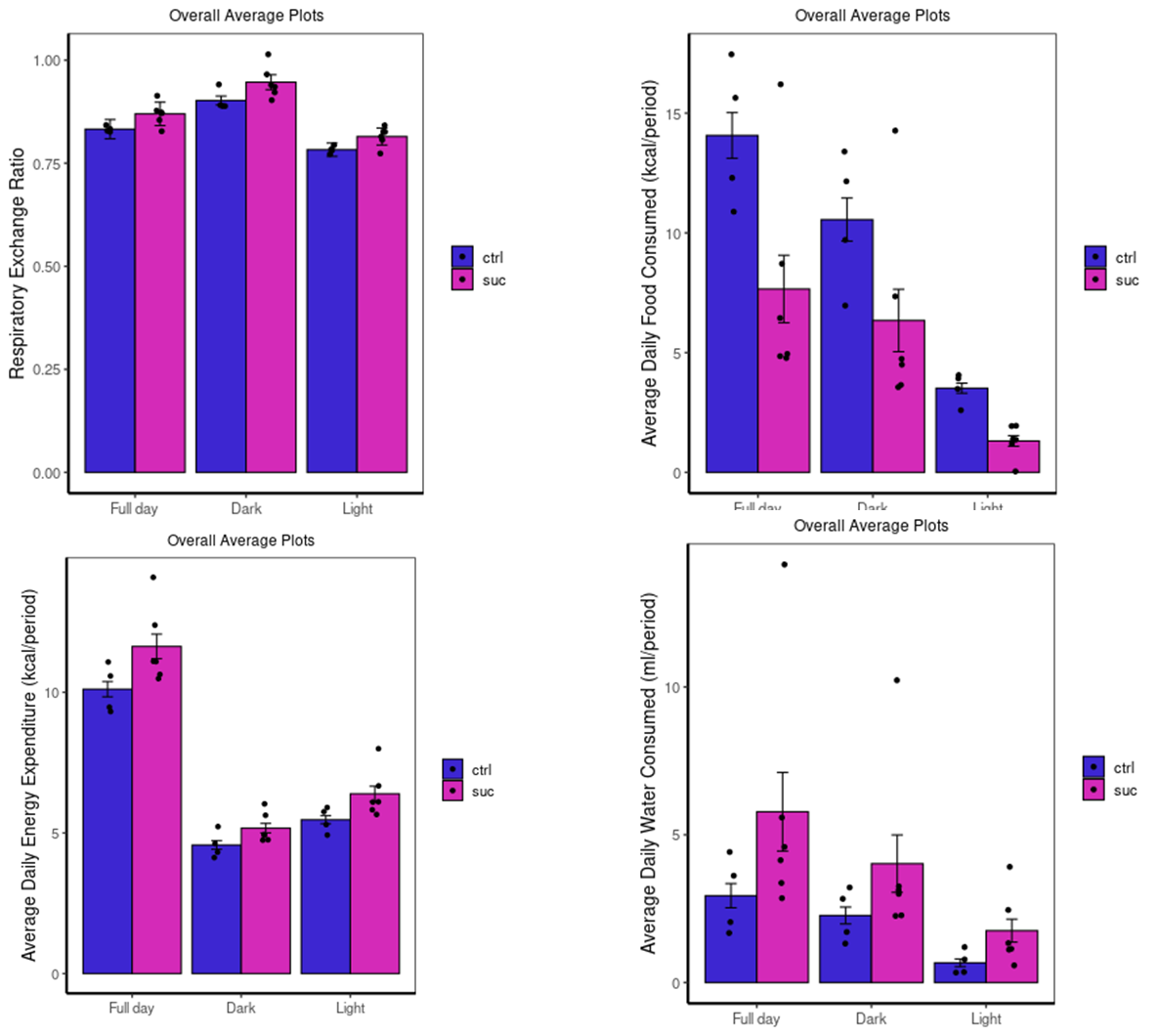
**Supplemental Figures:**

**Supplemental Figure 1.** Metabolic parameters of the mice were measured by the Comprehensive Laboratory Animal Monitoring System (CLAMS) using Promethean (Sable Systems). Following 48 hrs of acclimation, data were collected for 48 hrs. The data were analyzed using Expedata software system (v1.9.27), Macro interpreter (v2.40), and then CalR software (v.1.2). Values are means ± SEM.


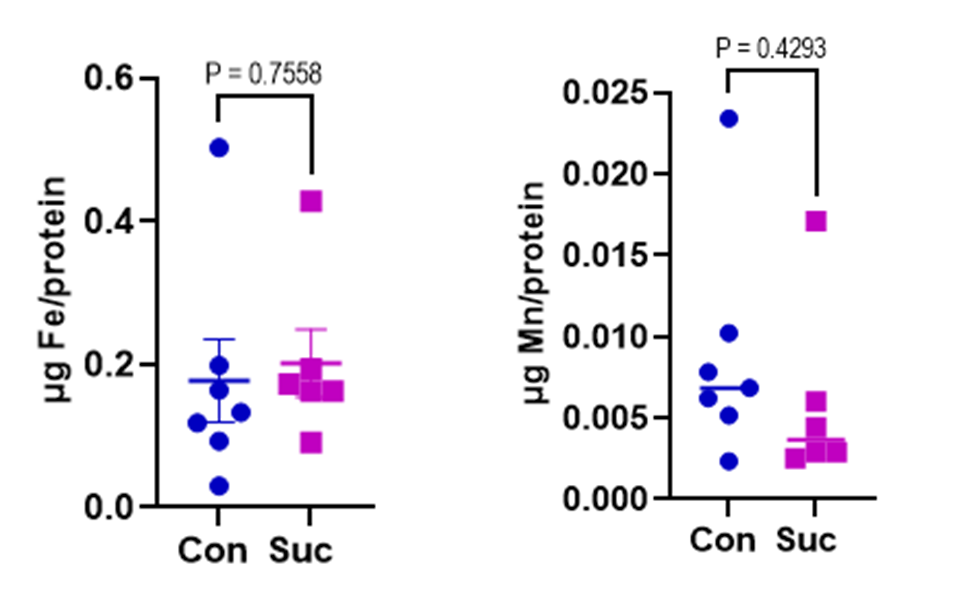


**Supplemental Figure 2. Fe and Mn levels in the isolated enterocytes**. Iron (Fe) and manganese (Mn) concentrations in intestinal epithelial cells (IEC) using MP-AES. IECs were separated and digested in nitric acid. Total protein concentrations were used for normalization. Values are means ± SEM; N= 4-7. Unpaired t-test between control and sucrose-treated groups.

**
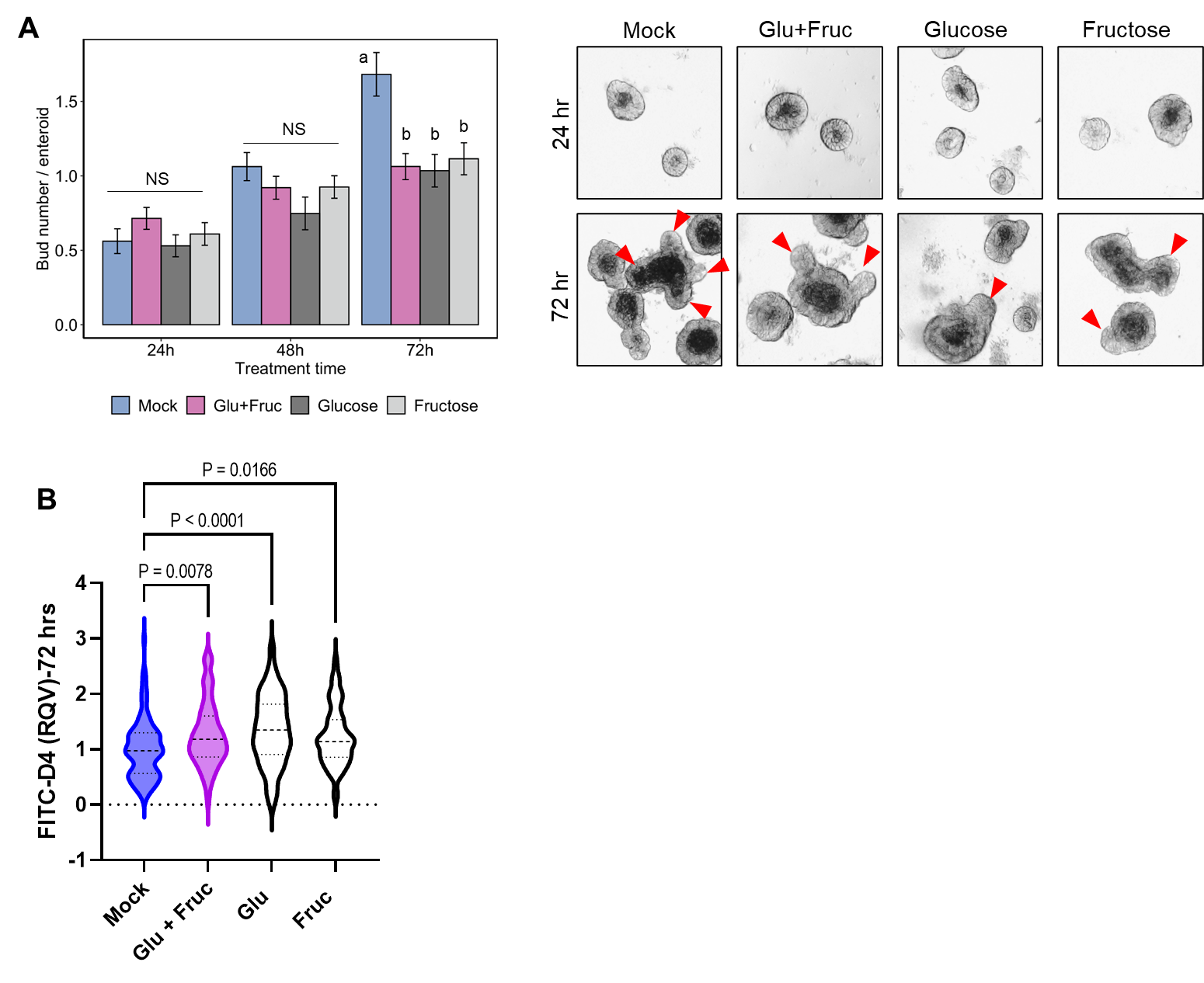
Supplemental Figure 3.** A) Bud numbers of enteroids at 24, 48, 72 hrs. post glucose alone (G), fructose alone (F), or cotreatment of G and F (GF) were quantified using the bright field images taken by OLYMPUS IX71 inverted microscope. Values are means ± SEM. . B) Following 72 hrs of Glucose (G), Fructose (F), and GF treatments, FITC-Dextran (green) fluorescence intensity within the enteroid was quantified by software ImageJ. Values are means ± SEM; # of enteroid counted: Mock, n = 102; GF, n = 92; G, n = 103; F, n = 102.

**
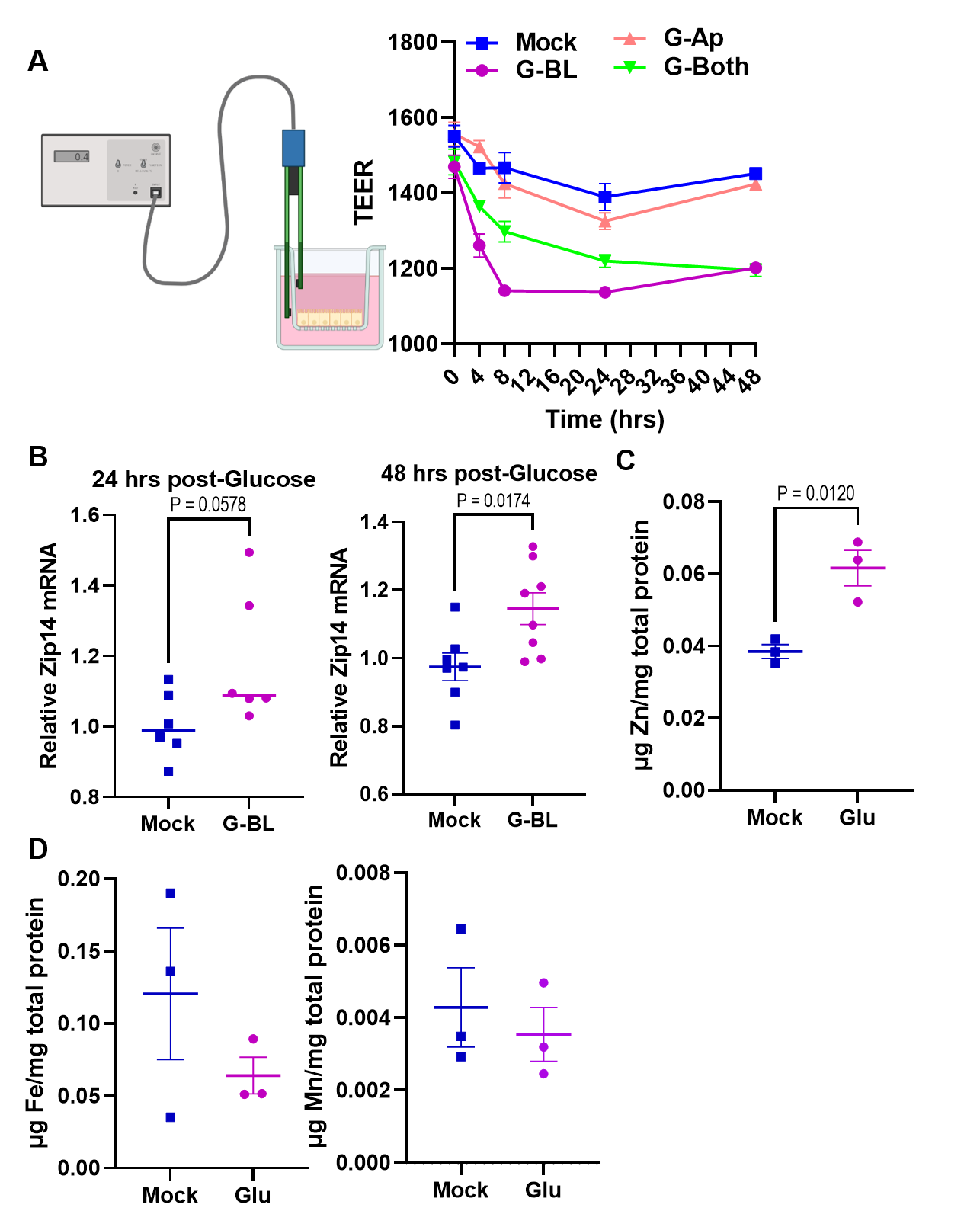
Supplemental Figure 4. High-glucose treatment increases permeability in Caco2 cells. A)** Caco2 cells were grown in a collagen-coated transwell system until full confluency. After 21 days at confluency, cells were treated with high glucose either basolaterally (G-BL), apically (G-Ap) or both (G-Both) for up to 48 hrs. Membrane resistance was measured, and changes in TEER values were plotted with time. B) At 24 and 48 hrs. post-glucose treatment, cells were harvested, RT-qPCR measured *zip14* mRNA levels, and relative fold changes of gene expression levels were analyzed. C, D) At 24 hrs. post glucose treatment, cells were harvested. Zn (C) and Fe and Mn (D) concentrations were determined by MP-AES. Values are expressed as the mean ± SEM. Unpaired t-test between control and sucrose-treated groups.

**
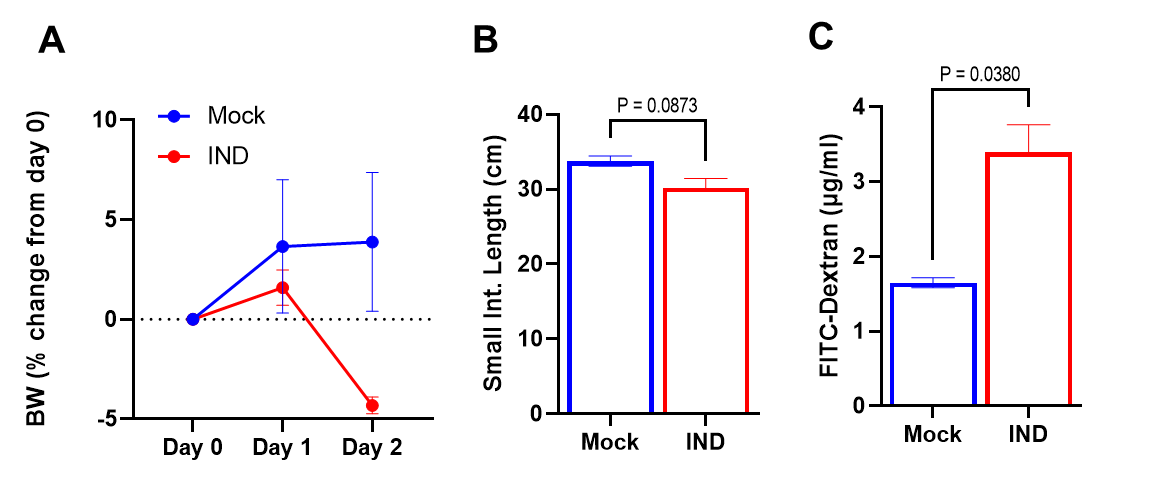
**

**Supplemental Figure 5. Indomethacin injections increase intestinal permeability.** Mice were injected (5 mg/ kg body weight) with indomethacin for two consecutive days. A) Percent body weight changes were calculated based on baseline weight. B) Length of small intestines was measured at the time of tissue collection. C) Following 2 days of indomethacin injection, mice were given FITC-Dextran after morning fasting. Blood was collected after two hours. FITC-Dextran concentrations in plasma were measured fluorometrically. Values are expressed as the mean ± SEM. Unpaired t-test between control and sucrose-treated groups.
